# Effects of transcranial alternating current stimulation on neurophysiologic motor function in Parkinson’s patients: a systematic review and meta-analysis

**DOI:** 10.3389/fnagi.2025.1621052

**Published:** 2025-09-03

**Authors:** Fangcheng Ye, Yinjin Shao, Guihua Wu, Miao Huang, Hui Huang

**Affiliations:** ^1^Department of Rehabilitation, Ganzhou People’s Hospital, Ganzhou, China; ^2^Ganzhou Key Laboratory of Neurological Rehabilitation, Ganzhou, China

**Keywords:** transcranial alternating current stimulation, Parkinson’s disease, rehabilitation therapies, motor evoked potentials, short-interval intracortical inhibition

## Abstract

**Objective:**

This systematic review and meta-analysis aimed to evaluate whether tACS improves neurophysiologic motor function in patients with Parkinson’s patients.

**Methods:**

We searched PubMed, Embase, Web of Science, and Cochrane Library for eligible studies from inception to March 2025. Measured outcomes included two indicators of neurophysiologic function: motor evoked potentials and short-term intracortical inhibition. The Cochrane risk of bias tool was used to evaluate the quality of the included literature, and extracted data were qualitatively synthesized and meta-analyzed.

**Results:**

Out of the 145 studies identified from the electronic databases, 7 fulfilled the inclusion criteria. Our results indicate that tACS significantly improved motor function in patients with PD compared to patients without tACS treatment. Motor function was assessed using motor evoked potentials (standardized mean deviation [SMD] = 2.65; 95% confidence interval [CI] = 2.02 to 3.27, *I*^2^ = 39%, *p* < 0.00001) and short-interval intracortical inhibition (SMD = 1.88; 95% CI = 1.47 to 2.30, *I*^2^ = 47%, *p* < 0.00001).

**Conclusion:**

Our findings suggested that tACS was strongly associated with improvements in motor evoked potentials and short-interval intracortical inhibition and could significantly improve neuromotor function. The results of this study provide additional evidence for the effectiveness of tACS and encourage the use of tACS in PD rehabilitation in clinical practice.

**Systematic review registration:**

The study protocol is registered with the International Prospective Register of Systematic Reviews under the registration number CRD420251016245.

## 1 Introduction

Parkinson’s disease (PD) is a progressive neurodegenerative disorder primarily characterized by the loss of dopaminergic neurons in the pars compacta of the substantia nigra, leading to cardinal motor symptoms including bradykinesia, rigidity, tremor, and postural instability (Poewe et al., 2017). Despite advancements in pharmacological and surgical treatments, a substantial proportion of patients persistently experience motor impairments that substantially compromise quality of life (Schapira et al., 2017). Consequently, there is a growing interest in non-invasive brain stimulation techniques, such as transcranial alternating current stimulation (tACS), as potential therapeutic interventions for motor dysfunction in PD (Zhang et al., 2022).

tACS is a non-invasive neuromodulation technique that delivers sinusoidal electrical currents through scalp electrodes to entrain cortical activity (Wischnewski et al., 2023). In contrast to transcranial direct current stimulation (tDCS) that employs a constant current, tACS generates frequency-specific oscillations (e.g., β-band at 13–30 Hz), thereby enabling the entrainment of neural oscillations and potential normalization of pathological brain rhythms underlying neurological disorders (Ghobadi-Azbari et al., 2021). In PD, excessive β-band (13–30 Hz) oscillatory activity within the basal ganglia-thalamocortical circuit has been established as a key pathophysiological mechanism contributing to motor symptoms (Gao et al., 2020). By targeting these aberrant oscillations, tACS holds promise as a novel therapeutic approach to ameliorate motor deficits in PD patients.

The rationale for using tACS in PD is further supported by evidence from animal models and human studies demonstrating the role of oscillatory activity in motor control (Lee et al., 2022). For instance, studies have shown that excessive beta-band synchronization in the basal ganglia-thalamocortical loop is associated with bradykinesia and rigidity in PD (Spooner and Wilson, 2023). Targeted modulation of these oscillations via tACS may disrupt pathological synchronization, thereby ameliorating motor dysfunction (Lafleur et al., 2021). Additionally, tACS has been shown to enhance neuroplasticity, which may contribute to its therapeutic effects (Elyamany et al., 2021). Computational models (e.g., Kuramoto oscillators) also suggest tACS may disrupt pathological β-band synchrony through phase-locking interference (Karimi et al., 2024). This phase interference is particularly applicable to the excessive beta synchronization observed in the basal ganglia-thalamo-cortical loop of PD patients. These findings underscore the potential of tACS as a non-invasive, targeted intervention for motor symptoms in PD.

Building upon these mechanistic foundations, tACS offers distinct practical advantages for clinical implementation in Parkinson’s disease management. Unlike other neuromodulation techniques requiring specialized facilities, tACS systems are portable and cost-effective, with recent technological innovations enabling simplified operation through pre-programmed stimulation protocols and quick-apply electrode arrays (Greenwald et al., 2025). Clinical studies have consistently demonstrated excellent tolerability, with fewer than 5% of participants discontinuing treatment due to minor side effects like transient scalp irritation (Liu et al., 2025). These features, combined with emerging evidence of sustained therapeutic effects, position tACS as a viable candidate for decentralized Parkinson’s care – potentially enabling community health centers or even home-based treatment under remote monitoring.

Despite increasing preclinical and clinical investigations, the therapeutic efficacy of tACS for motor dysfunction in PD has not been definitively established. Preliminary studies have demonstrated tACS-induced improvements in bradykinesia, gait, and fine motor control, though these findings are limited by small sample sizes and methodological heterogeneity (Del Felice et al., 2019; Guerra et al., 2023b). However, these studies are often limited by small sample sizes, heterogeneous methodologies, and a lack of long-term follow-up (Fregni et al., 2021). Moreover, the optimal stimulation parameters, including frequency, intensity, and duration, remain poorly defined (Antal et al., 2017). Given these uncertainties, a systematic review and network meta-analysis are warranted to synthesize the available evidence and provide a comprehensive assessment of the effects of tACS on motor function in PD.

The aim of this systematic review and meta-analysis is to assess the efficacy of tACS in improving motor function in patients with Parkinson’s disease. By integrating data from randomized controlled trials (RCTs), this review will provide a strong evidence base to guide clinical practice and future research. In addition, the findings of this review will have a significant impact on the development of non-invasive therapeutic strategies for Parkinson’s disease and contribute to the growing body of literature on neuromodulation in neurodegenerative diseases.

## 2 Methods

This systematic review and meta-analysis was conducted in accordance with the PRISMA (Preferred Reporting Items for Systematic Reviews and Meta-Analyses) guidelines (see Supplementary Table S1) (Page et al., 2021). The study protocol is registered with the International Prospective Register of Systematic Reviews under the registration number CRD420251016245 on Mar 21, 2025.

### 2.1 Search strategy

Two authors (FY and MH) independently searched four different electronic databases, including PubMed, Web of Science, EMBASE, and Cochrane Library, for eligible articles from inception to January 2025. The following terms were used for electronic searching: (“transcranial alternating current stimulation” [Title/Abstract] OR “tACS” [Title/Abstract]) AND (“Parkinson Disease” [MeSH Terms] OR (“idiopathic Parkinson’s disease” [Title/Abstract] OR “Lewy body Parkinson’s disease” [Title/Abstract] OR “Parkinson’s disease idiopathic” [Title/Abstract] OR “Parkinson’s disease Lewy body” [Title/Abstract] OR “paralysis agitans” [Title/Abstract] OR “Parkinson’s disease” [Title/Abstract] OR “idiopathic Parkinson disease” [Title/Abstract] OR “Lewy body Parkinson disease” [Title/Abstract] OR “primary parkinsonism” [Title/Abstract] OR “parkinsonism primary” [Title/Abstract] OR “Parkinson disease idiopathic”[Title/Abstract])). Full search syntax is provided in Supplementary Table S2. The keywords were used with the PubMed filter and selected using medical subject headings. The search terms were adapted for use with the other electronic databases. Included study references and clinical trial registries were hand-searched. There was no publication date, age, or setting restrictions; however, only articles published in English were included.

### 2.2 Selection criteria

Two authors (FY and GW) independently screened the titles, abstracts, and full texts to identify eligible studies for inclusion in this systematic review and meta-analysis. Studies were considered to include if they met the following criteria: (1) Patients diagnosed with Parkinson’s disease without comorbid neurological disease by a clinician or using any recognized diagnostic criteria; (2) The intervention group was transcranial alternating current therapy; (3) included a comparator group comprising PD patients who received sham tACS, standard care, placebo, or other rehabilitative therapies excluding tACS; (4) were a clinical randomized control trial, quasi RCT, crossover RCT study, or comparative study; and (5) were published in English.

Studies were considered excluded if they: (1) were a preclinical study; (2) had no control group; (3) were conference abstracts, communications, a letter with no empirical data, or commentary; or (4) did not include the full text.

### 2.3 Risk of bias and quality assessment

Risk of bias was independently assessed by two reviewers using the Cochrane Collaboration’s risk of bias tool (Higgins et al., 2011). The risk of bias tool covers six domains of bias: selection bias, performance bias, detection bias, attrition bias, reporting bias, and other bias. The second part of the tool involves assigning a judgment of high, low, or unclear risk of material bias for each item. The result was independently rated by two authors (FY and YS). We read the study designs of the included articles in detail, and we defined them as randomized controlled studies as long as the articles detailed the randomization method or explained it in the experimental design. We defined articles as non-double-blind studies if they had a non-double-blind statement or explained non-double-blindness during the experimental procedure, otherwise all were considered to have reached the double-blind level. Any disagreements on the risk of bias or quality assessments were resolved by a third author or the research team.

### 2.4 Data extraction

Data extraction was conducted independently by two reviewers (HH and MH) using a standardized piloted form, with discrepancies resolved through consensus or third-party adjudication. Any discrepancies that arose during this process were resolved through discussion. The following data elements were extracted from the included studies: (1) study source (authors, publication year), (2) participant information (number of participants in each group, mean age, sex, Medication status (ON/OFF levodopa during tACS), Unified Parkinson’s Disease Rating Scale (UPDRS III) motor component score, Hoehn & Yahr stage, Montreal Cognitive Assessment (MoCA) score, Beck Depression Inventory-II, disease duration, Daily dose of levodopa), (3) interventions, and (4) outcome measures.

For continuous outcomes, mean ± standard deviation (SD) values were extracted. When unavailable, data were derived from standard errors, confidence intervals, or medians using Cochrane Handbook conversion methods. If means and SD were not provided in the included studies, data presented in the form of standard errors, confidence intervals, or medians with ranges were converted to mean and standard deviation format using established statistical formulas recommended in the literature. For missing data in the articles, the authors of the article were contacted for detailed data.

### 2.5 Data synthesis and analysis

All the data review and meta-analysis were performed using the Review Manager 5.3 (Version 5.3; Cochrane, Oxford, United Kingdom). The difference between the control group and the intervention group was estimated. Continuous variable data were selected for the standardized mean difference (SMD) analysis. Each effect volume was expressed as a 95% confidence interval (CI). The heterogeneity among the results of included studies was determined based on values of *Q* and *I*^2^ statistics (Migliavaca et al., 2022). *I*^2^ ≤ 50% indicated homogeneity between the studies, which was calculated using the fixed-effects model. *I*^2^ > 50% indicated heterogeneity between studies, and a random-effects model was used instead (Higgins and Thompson, 2002). Subgroup analyses were planned *a priori* to explore heterogeneity sources. We also assessed publication offsets by visually inspecting funnel plots (Page et al., 2021).

## 3 Results

### 3.1 Study identification

**Figure 1:**
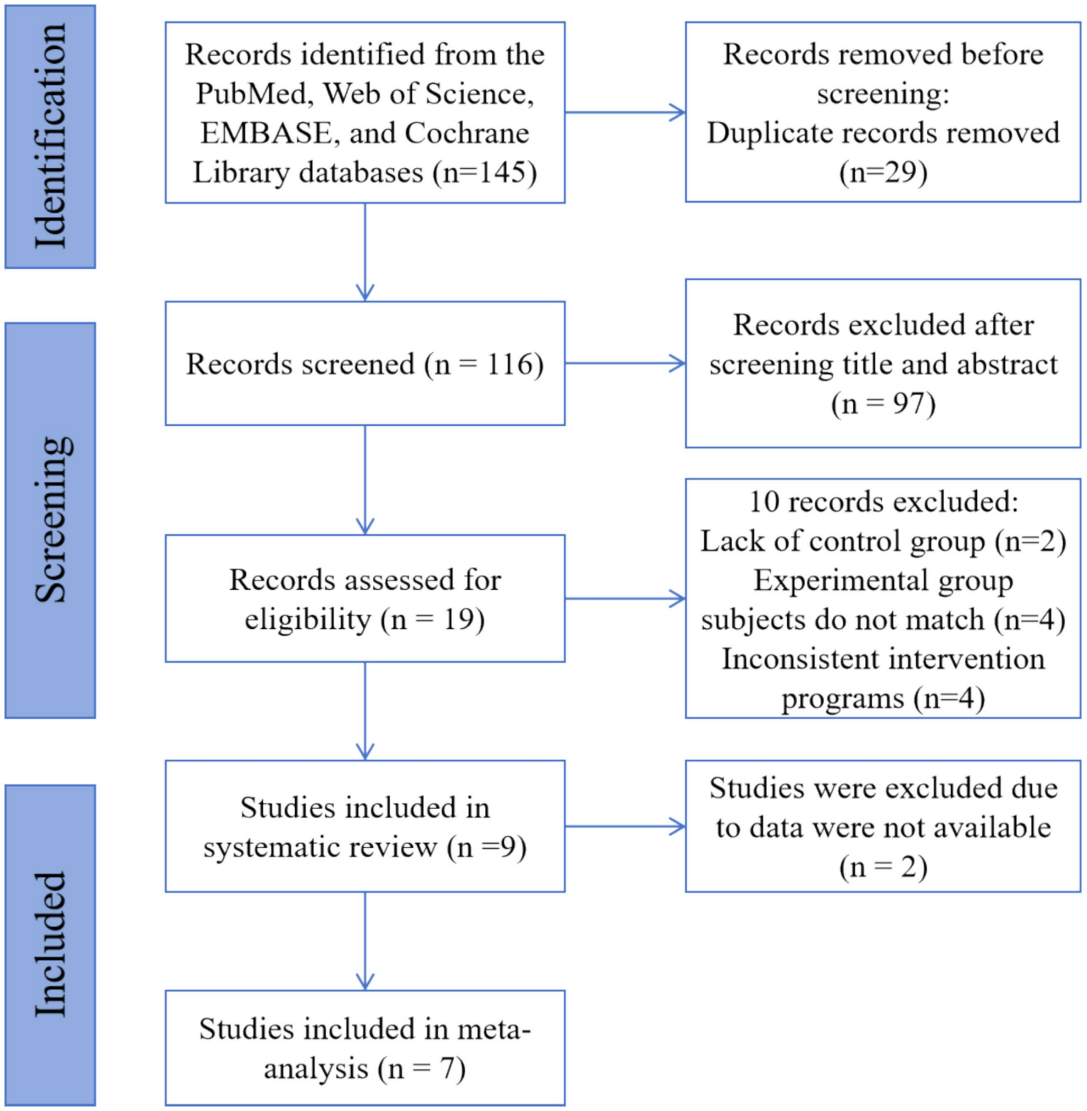
PRISMA flow diagram for identifying eligible studies.

The systematic search identified 145 unique records from PubMed, Web of Science, EMBASE, and Cochrane Library (PRISMA flow diagram, Figure 1). We then screened titles and abstracts of 116 records after removing 29 duplicates. Altogether, 97 records were excluded. Then, we evaluated the full text of 19 records. After the full-text reading, it is found that 10 texts did not meet the inclusion criteria: 2 records were lacking a control group, 4 records had mismatched subjects in the experimental group, and 4 others had inconsistent intervention programs. Overall, 9 studies were eligible and were enlisted in this systematic review. All studies were RCTs. Since two studies were not able to extract appropriate data, a meta-analysis was performed from the data of 7 studies.

### 3.2 Study characteristics

The demographic characteristics, intervention and comparator descriptions, and outcome measures are illustrated in Table 1. The final analysis included 173 participants (male: 65.3%, *n* = 113; female: 34.7%, *n* = 60). The mean age ranged from 45 to 79 years. Among all participants included, the mean duration of Parkinson’s disease ranged from 0.3 to 11.3 years, the patient’s Hoehn & Yahr stage was basically 1–2, the daily L-Dopa dose ranged from 142 mg to 859.5 mg, and UPDRS III scores ranged from 9.5 to 53.3. In addition to this, five studies reported MoCA scores and four reported BDI-II.

**Table 1:** The characteristics of included studies in the systematic review.

Author (year)	Sample size (con/exp)	Ages (years)	Disease duration (years)	Gender (men/women)	Medication status	daily L-Dopa dose (mg)	Hoehn and Yahr	UPDRS III	MoCA	BDI–II	Intervention	Outcome measures
Krause et al. (2013)	20 (10/10)	48.6 ± 11.6	1.9 ± 1.6	20 (10/10)	ON	270.9 ± 123.7	1–2	19.8 ± 10.1	–	–	tACS	CMC
Del Felice et al. (2019)	15 (11/4)	69 ± 6.3	6.3 ± 4.8	15 (9/6)	–	528.5 ± 290	1–2	33 ± 12.3	23.3 ± 2.1	7.9 ± 5.8	tACS	UPDRS III, MoCA
Guerra et al. (2020)	32 (16/16)	67.2 ± 9.8	5.6 ± 3.2	32 (21/11)	OFF	540.3 ± 227.7	1.7 ± 0.5	29.1 ± 13.3	26.9 ± 2.4	6.7 ± 4.5	tACS	MEP, SICI
Guerra et al. (2022)	34 (18/16)	66.9 ± 9.7	6.8 ± 3.6	34 (23/11)	OFF	525.6 ± 333.9	–	33.6 ± 9.3	26.9 ± 2.3	8.7 ± 5.6	tACS	MEP, SICI
Guerra et al. (2023a,b)	27 (14/13)	66.9 ± 9.4	5.1 ± 2.8	27 (20/7)	OFF	–	–	22.9 ± 9.7	27.5 ± 2.3	–	tACS	MEP, SICI
Guerra et al. (2023a,b)	30 (15/15)	66.2 ± 9.9	6.1 ± 3.4	30 (23/7)	OFF	436 ± 176	–	33.5 ± 10.9	27.6 ± 1.9	8.1 ± 5.9	tACS	MEP, SICI
Benussi et al. (2024)	14 (7/7)	73.1 ± 5.9	4.3 ± 2.3	14 (7/7)	ON	–	2.4 ± 0.9	32.6 ± 13.6	–	–	tACS	MEP, SICI

tACS, transcranial alternating current stimulation; CMC, Cortico-muscular coupling; UPDRS III, Unified Parkinson’s Disease Rating Scale; MoCA: Montreal Cognitive Assessment; MEP, motor evoked potential; SICI, Short-interval intracortical inhibition.

Of the seven studies included in the systematic review (Del Felice et al., 2019; Guerra et al., 2023b; Krause et al., 2013; Guerra et al., 2022; Guerra et al., 2023a; Guerra et al., 2020; Benussi et al., 2024), four explicitly used γ-tACS and the other three did not specify (Guerra et al., 2023b; Guerra et al., 2022; Guerra et al., 2023a; Guerra et al., 2020). Among primary outcomes, motor evoked potential (MEP) and short-interval intracortical inhibition (SICI) were the most reported (5 studies each). Only one study reported results for UPDRS III (Del Felice et al., 2019). In the other, there was one study that included cortical-muscle coupling as a primary outcome.

### 3.3 Risk of bias and quality of included studies

**Figure 2:**
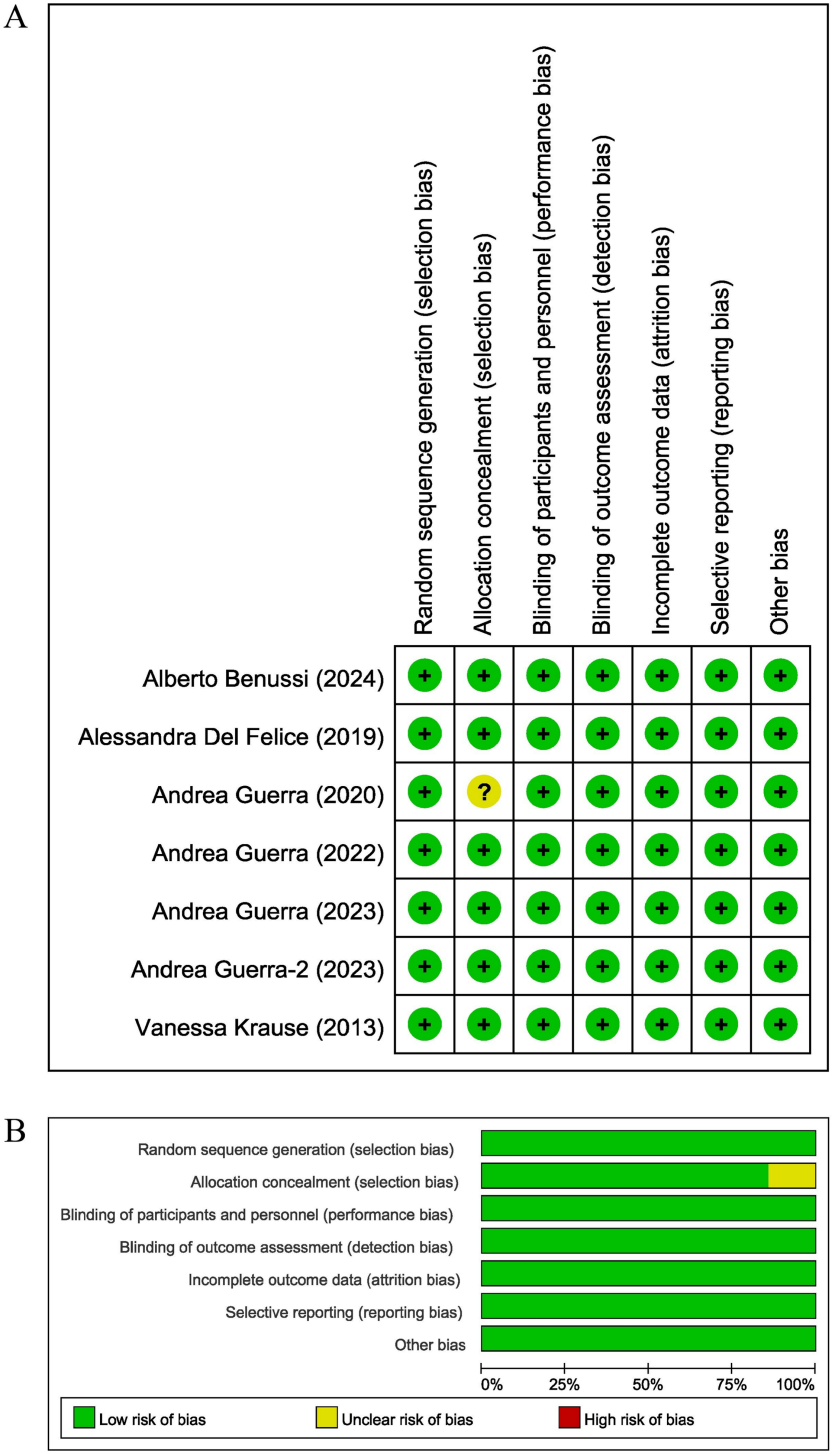
The result of the risk of bias assessment. **(A)** Risk of bias graph; **(B)** Risk of bias summary.

Figure 2 shows the risk of bias for the included randomized controlled studies. The results showed that the positional concealment technique was unclear in only one study, and the risk of bias was low in each of the other included studies. This indicates that the level of evidence for all seven studies was high.

### 3.4 Primary outcome measures

Not all studies chose the same primary outcome assessment. The following outcomes were assessed: MEP and SICI. In the five included studies, electrophysiologic signals recorded with peripheral muscles or nerves in response to MEP, in mV (Guerra et al., 2023b; Guerra et al., 2022; Guerra et al., 2023a; Guerra et al., 2020; Benussi et al., 2024). In addition, these five articles assessed the results of short-interval intracortical inhibition in patients in response to motor cortex inhibitory functions. In addition to these studies, a small number of studies used cortico-muscular coupling, UPDRS III scores, MoCA scores and MMSE as an assessment of treatment efficacy.

### 3.5 Motor evoked potential

**Figure 3:**
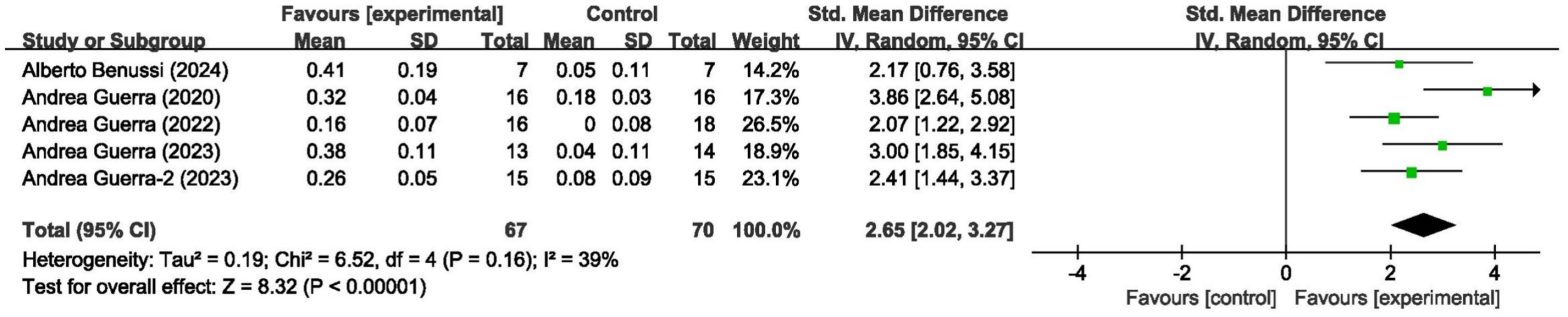
Forest plot showing the effects of tACS on MEP.

These 5 studies provided data on a total of 137 participants, 70 in the control group and 67 in the intervention group. Meta-analysis of MEP amplitude (5 studies, *n* = 137) demonstrated a large effect size favoring tACS (SMD = 2.65, 95% CI: 2.02–3.27; *I*^2^ = 39%, indicating low heterogeneity; *p* < 0.00001) (Figure 3).

### 3.6 Short-interval intracortical inhibition

**Figure 4:**
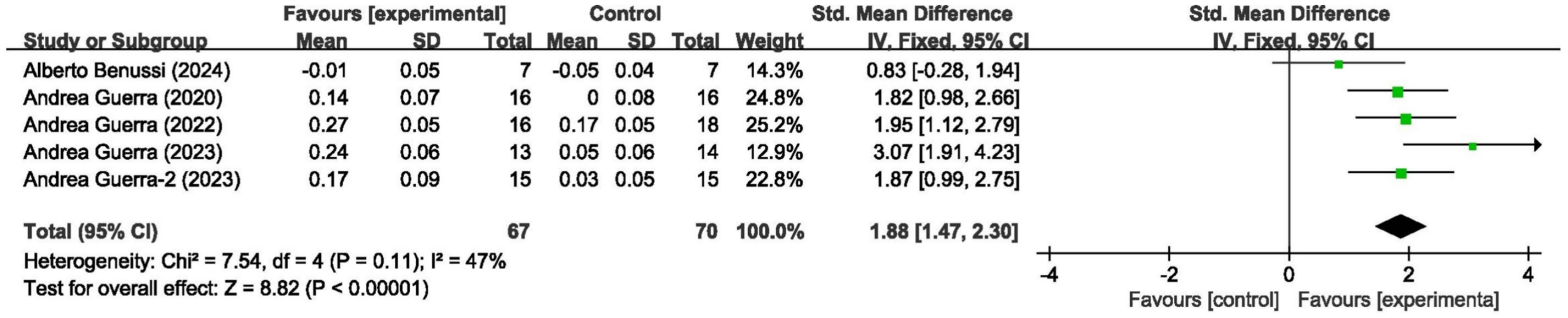
Forest plot showing the effects of tACS on SICI.

For short-interval intracortical inhibition, pooled data from 5 studies (*n* = 137; intervention: *n* = 67, control: *n* = 70) showed significant improvement (SMD = 1.88, 95% CI: 1.47–2.30; *I*^2^ = 47%, *p* < 0.00001) (Figure 4).

### 3.7 Subgroup analyses

**Figure 5:**
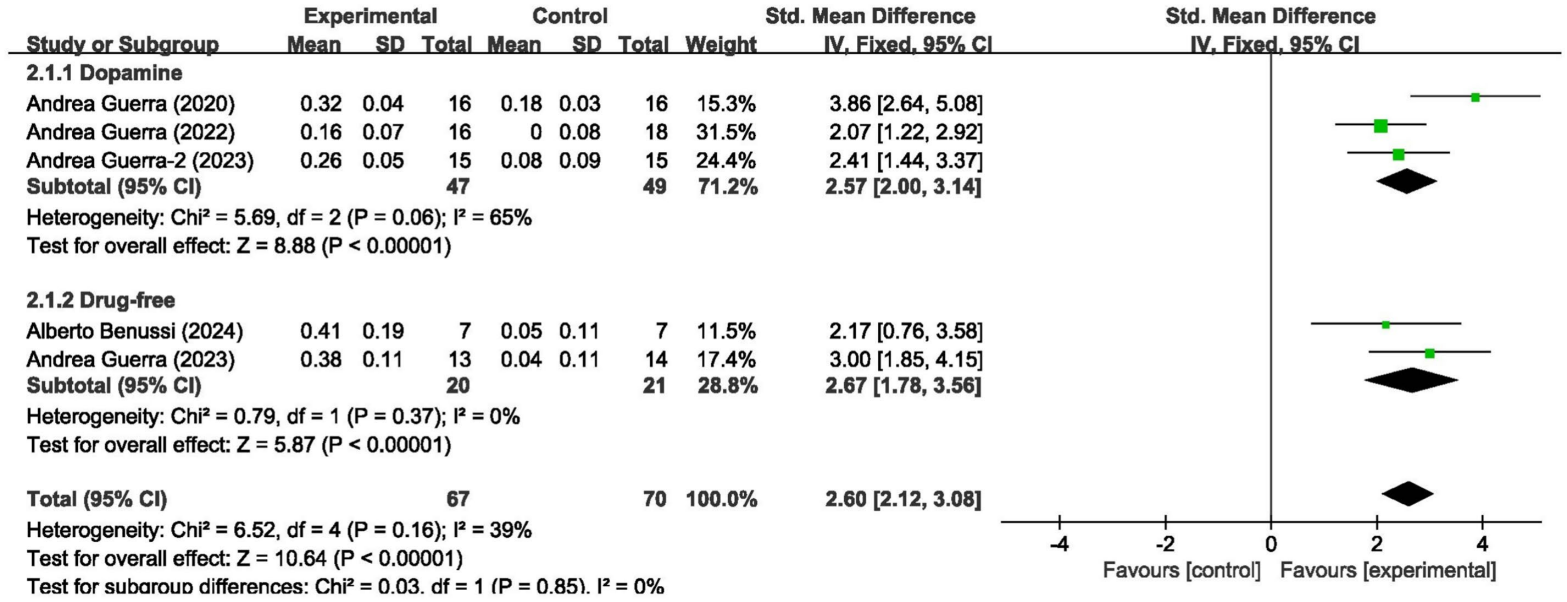
Forest plot showing the effect of L-dopa medication administration on MEP.

**Figure 6:**
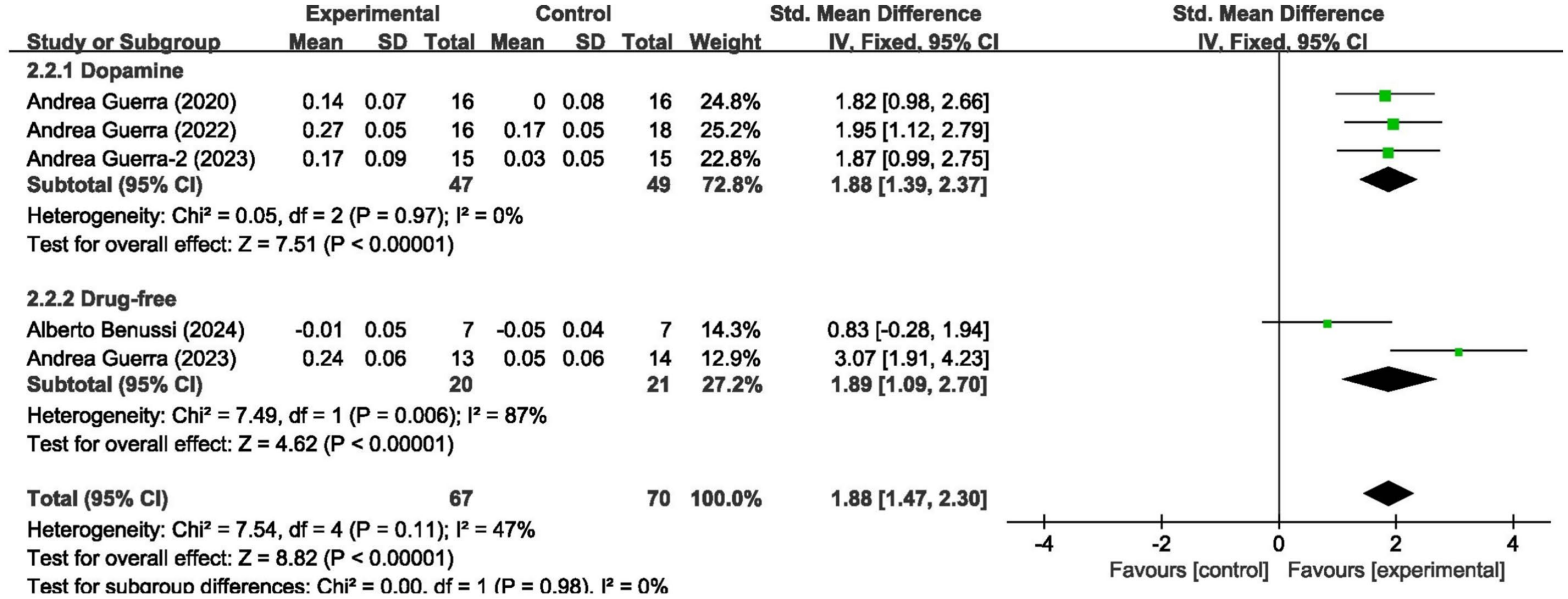
Forest plot showing the effect of L-dopa medication administration on SICI.

**Figure 7:**
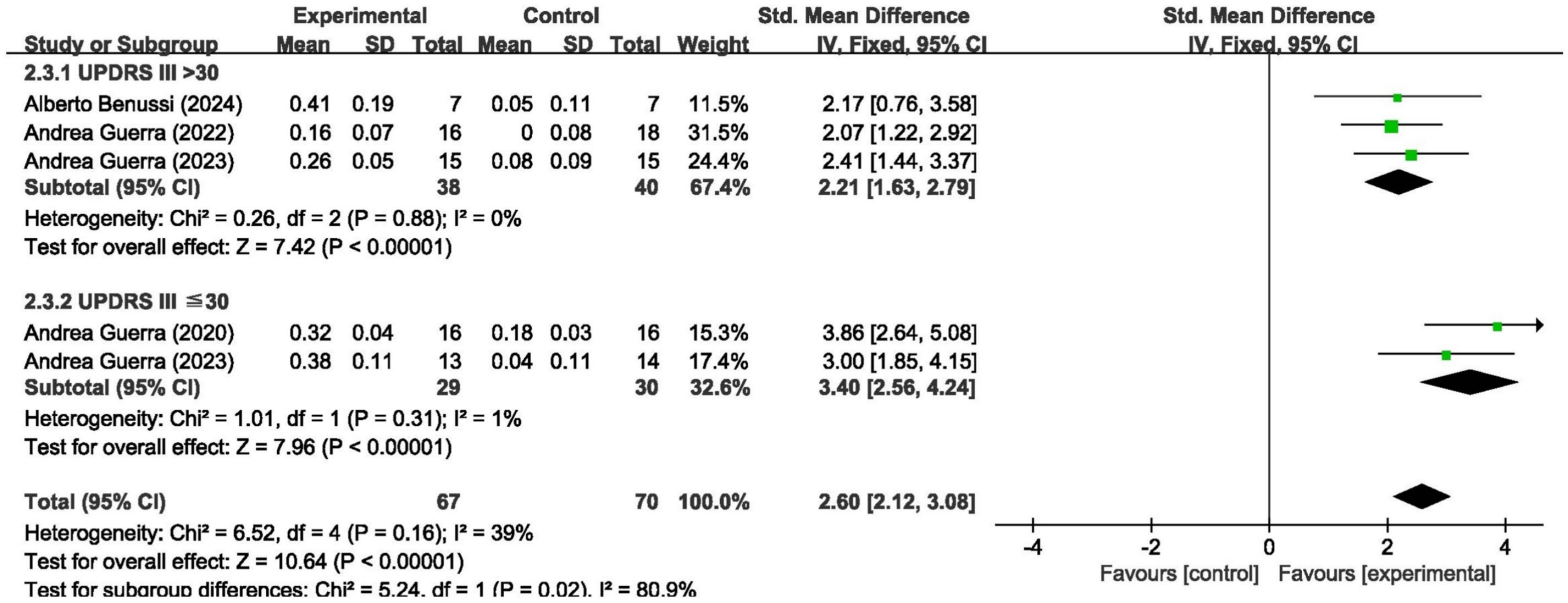
Forest plot showing the effect of baseline UPDRS III scores on MEP.

**Figure 8:**
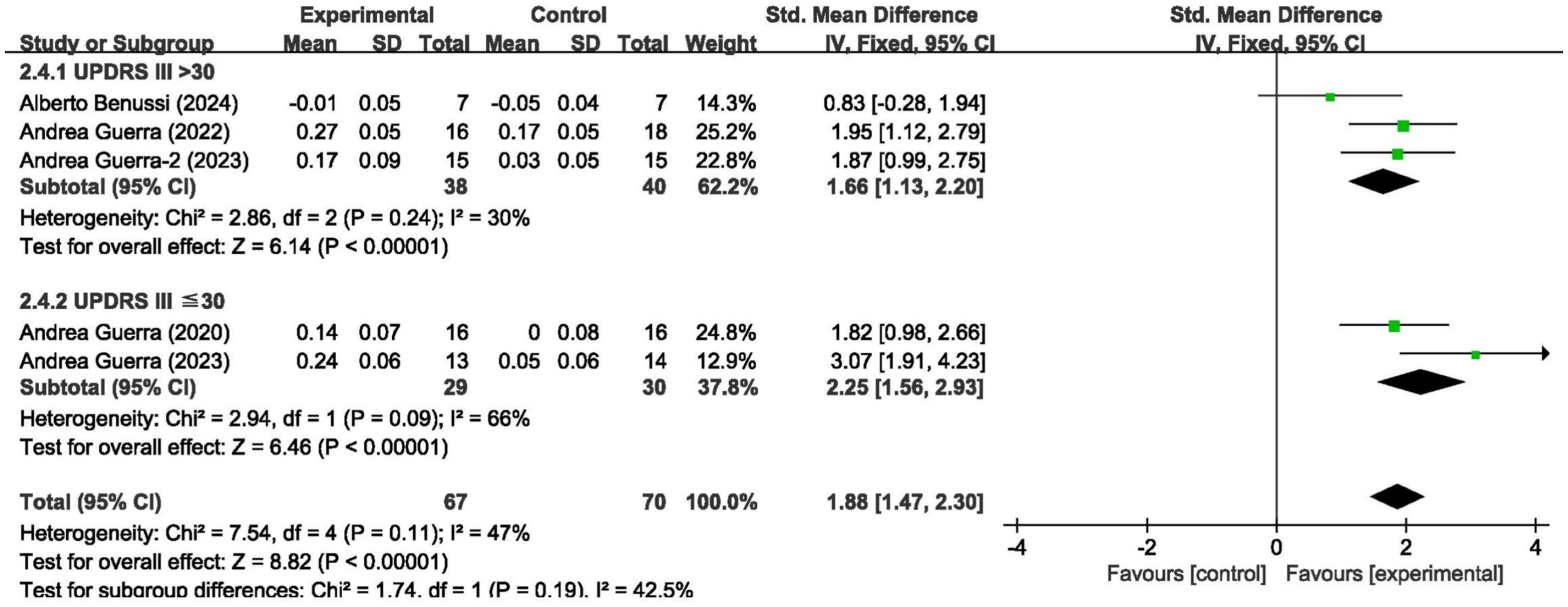
Forest plot showing the effect of baseline UPDRS III scores on SICI.

We analyzed the results in subgroups to examine the factors affecting efficacy. Among other things, we verified that the presence or absence of L-dopamine was the main factor influencing efficacy. The final results showed that MEP changes were more significant in PD taking dopamine (SMD = 2.57, 95% CI = 2.00 to 3.14, *I*^2^ = 65%, *p* < 0.001) than in those not receiving dopamine (SMD = 2.67, 95% CI = 1.78 to 3.56, *I*^2^ = 0%, *p* < 0.001). No significant subgroup difference (*p* = 0.82). In addition, we found that changes in SICI were more pronounced when taking dopamine (SMD = 1.88, 95% CI = 1.39 to 2.37, *I*^2^ = 0%, *p* < 0.001) compared to changes in the absence of it (SMD = 1.89, 95% CI = 1.09 to 2.70, *I*^2^ = 87%, *p* < 0.001). The results are presented in Figures 5, 6. In addition, we considered that UPDRS III scores may be an important factor in the source of heterogeneity. Subgroup analyses showed that SICI heterogeneity was partially attributable to differences in baseline disease severity (UPDRS III > 30: *I*^2^ = 30%; UPDRS III ≤ 30: *I*^2^ = 66%). The results are presented in Figures 7, 8.

### 3.8 Publication bias and sensitivity analysis

**Figure 9:**
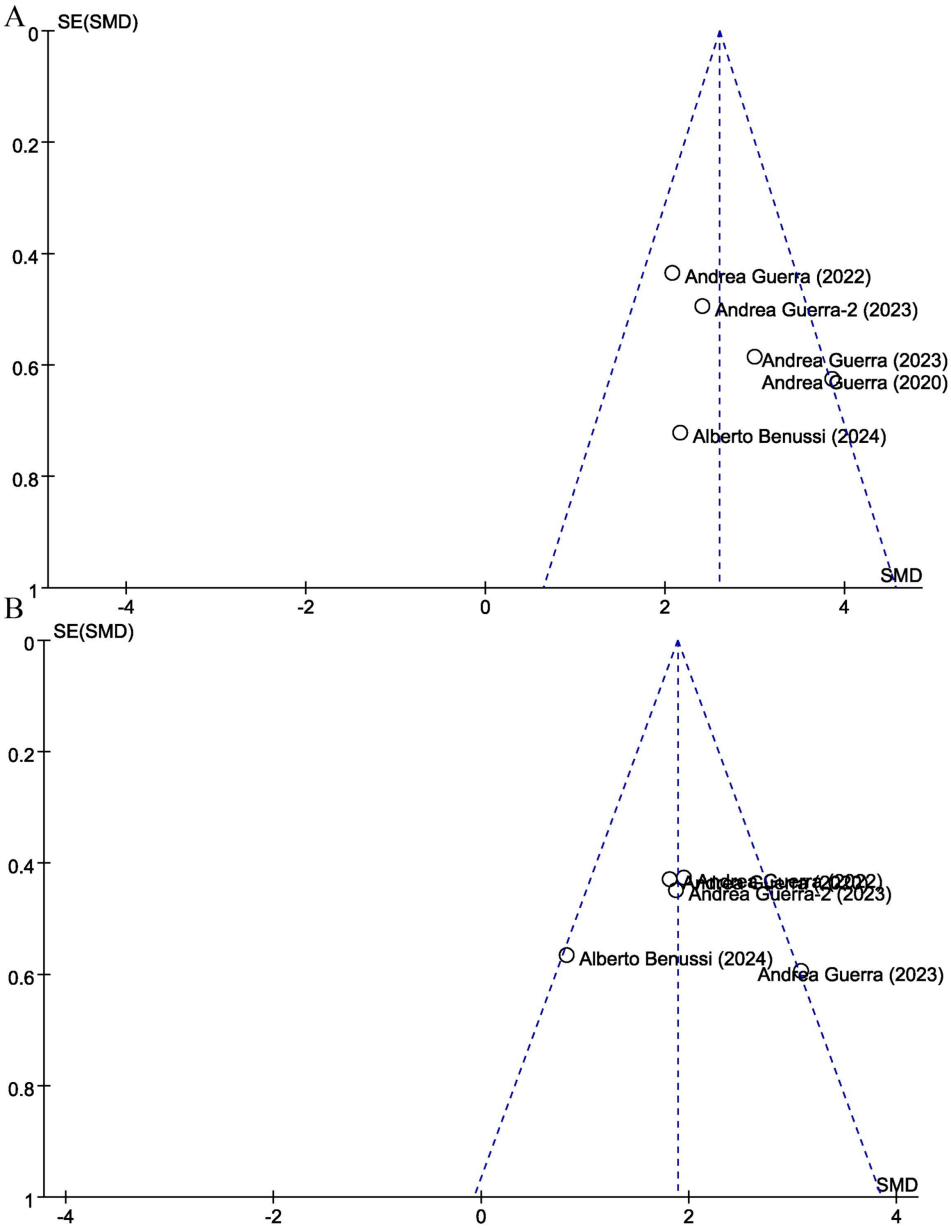
Funnel plots assessing publication bias for **(A)** motor evoked potentials (MEP) and **(B)** short-interval intracortical inhibition (SICI). Each point represents an individual study (labeled by first author and year).

Funnel plots revealed no evidence of publication bias for primary outcomes (Figure 9). Sensitivity analyses using alternative statistical models yielded consistent results.

## 4 Discussion

This meta-analysis of 7 randomized controlled trials (*n* = 173) provides Level I evidence that tACS significantly enhances motor function in Parkinson’s disease, particularly through modulation of corticospinal excitability (MEP) and intracortical inhibition (SICI). In addition, there was one study with the same aim as ours, but this study lacked a control group and was therefore not included in the meta-analysis (Rahimi et al., 2023). In conclusion, the primary findings indicate that tACS significantly enhances MEP amplitude and SICI, suggesting improved corticospinal excitability and motor cortical plasticity. The superior treatment response in dopaminergic medication users (SMD = 2.57 vs. 2.67) suggests a synergistic mechanism, possibly through tACS-induced enhancement of striatal dopamine receptor sensitivity.

Brain stimulation techniques are considered when medications fail to adequately relieve clinical symptoms in Parkinson’s patients. Commonly used non-invasive brain stimulation paradigms include repetitive transcranial magnetic stimulation, tDCS, and tACS (Madrid and Benninger, 2021). To the best of our knowledge, available systematic reviews and meta-analyses have focused on the effects of tDCS on motor function in PD patients (Lee et al., 2019; Elsner et al., 2016; de Oliveira et al., 2021), and few reviews related to the effects of tACS on motor function in PD patients have been seen. To our knowledge, this is the first meta-analysis specifically evaluating tACS for PD motor symptoms. While the effect sizes (SMD 1.88–2.65) are comparable to tDCS/TMS studies, tACS offers unique frequency-specific neuromodulation absent in other techniques (Nguyen et al., 2024; Giustiniani et al., 2025).

Transcranial alternating current stimulates neural tissue by means of alternating current at a certain frequency. Unlike tDCS, it has the advantage that it allows modulation of the oscillatory activity of the neural network due to frequency stimulation at almost imperceptible current strengths (Birreci et al., 2024). The brain’s oscillatory activity at certain frequencies reflects the activation of specific sensorimotor functions (Herrmann et al., 2016). Therefore, attempts have been made to improve motor function in PD patients by enhancing or inhibiting current processes via tACS to increase or decrease the amplitude of oscillations.

Motor threshold has traditionally been defined as the lowest TMS stimulus intensity capable of eliciting a small MEP, and SICI denotes the greater conditioned test stimulus intensity required to generate and maintain the target MEP response, both of which can respond to changes in brain motor function (Vucic et al., 2023). In the present study, we found a significant increase in MEP amplitude in the experimental group of Parkinson’s patients after tACS intervention compared to the control group (SMD = 2.65, *p* < 0.00001), which suggests that tACS enhances the excitability of the corticospinal tracts. This result is consistent with the findings of Guerra et al. who found that γ-tACS improves motor function in Parkinson’s disease patients by restoring synaptic plasticity in the primary motor cortex (Guerra et al., 2023b). In addition, there was also a significant improvement in SICI in the experimental group after tACS intervention (SMD = 1.88, *p* < 0.00001).

In the early stages of Parkinson’s disease, L-dopamine is a very effective treatment for PD (Haddad et al., 2017). However, it is important to note that levodopa medication was not taken in the same way in these studies. Therefore, we performed a subgroup analysis assuming whether or not dopamine medication was taken as a source of heterogeneity in the data. PD patients in two studies were not taking dopamine medication, but their motor function improved equally significantly after the tACS intervention (Guerra et al., 2023a; Benussi et al., 2024). The results of the subgroup analysis suggested that dopamine medication use was indeed an important source of heterogeneity in this meta-analysis. In addition, the observed heterogeneity may reflect differences in the severity of Parkinson’s disease in different patients. Future studies should use uniform outcome assessments and stratify analyses by disease stage to minimize confounders.

With this in mind, we considered whether accurate tACS could be achieved through machine learning. Studies have shown that supervised learning of baseline UPDRS III scores and resting-state β-power predicts treatment response. For example, a randomized forest model integrating age and disease duration achieved 82% accuracy in predicting the outcome of transcranial magnetic stimulation, a framework applicable to tACS (Hamzyan Olia et al., 2025). In addition, closed-loop tACS, where stimulation is adjusted via real-time EEG classification of β-bursts, represents a transformative frontier for home-based therapy.

Of course, these included articles vary in their choice of tACS stimulation frequency, and it is not clear which stimulation frequency works best. They are mainly categorized as γ-tACS, β-tACS, and α-tACS, with different frequencies having different therapeutic effects on neurological disorders. Our data do not identify which frequency band has better efficacy, and the variation between studies highlights the need for adaptive trials. Bayesian optimization can efficiently navigate the parameter space (e.g., 10–30 Hz, 1–2 mA) to determine patient-specific protocols (frequency, intensity, duration, electrode montage). Many studies have shown that γ-tACS modulates neural oscillations, enhances cognitive function and provides neuroprotection (Sanchez-Garrido Campos et al., 2025). β-tACS may restore the balance of cortico-basal ganglia circuits by “desynchronizing” abnormal β oscillations (Naros and Gharabaghi, 2017). α-tACS may improve sensorimotor integration by modulating thalamo-cortical circuits (Fresnoza et al., 2020). β-γ PAC disruption may serve as a biomarker, with tACS selectively attenuating excessive phase-locking in the basal ganglia-thalamocortical loop (Salimpour et al., 2022). Perhaps different stimulation frequencies are also a source of heterogeneity, but the small amount of literature did not allow for further analysis. The variation in stimulation parameters (e.g., frequency, intensity) across studies highlights the need for dose mapping tests. Future work should systematically compare parameter sets to establish evidence-based protocols.

Current studies also have many limitations. First, the sample sizes of the included studies are small (median *n* = 25 per study), which may affect the validity of our results, as well as the generation of heterogeneity. While our findings are statistically significant, the limited number of studies restricts the generalizability of the conclusions. Future large-scale, multicenter RCTs with standardized protocols are urgently needed to validate these preliminary results and assess the long-term efficacy of tACS in PD. Second, differences in tACS parameters (e.g., frequency, duration of sessions) and control conditions (sham versus standard treatment) may confound results. Third, the measurement of outcomes lacks a uniform quantitative metric. Although MEP and SICI are valuable physiological metrics, they do not fully reflect the multidimensional nature of motor function in Parkinson’s disease (Washabaugh et al., 2024). MEP/SICI provide limited insight into real-world motor function. Combining wearable sensors with ML pipelines (e.g., convolutional neural networks to analyze tremor kinematics) could bridge this gap. Fourth, there is a lack of long-term follow-up data to assess persistence of efficacy. Finally, although the funnel plot showed minimal bias, the small number of studies made it difficult to draw definitive conclusions. Therefore, future trials should prioritize conducting long-term assessments and harmonizing metrics to validate tACS as a reliable adjunctive therapy. And, future studies should be reported with complete stimulation protocols, including electrode locations (10–20 systems), waveforms, impedance, and total charge, to improve replicability and for computational modeling.

## 5 Conclusion

By integrating evidence from seven randomized controlled trials, this study demonstrates for the first time that tACS significantly improves motor function in patients with Parkinson’s disease, with clear clinical significance in terms of effect size (SMD 1.88–2.65). Notably, when tACS was combined with dopaminergic drugs, improvements in MEP and SICI reached 265 and 188%, respectively, a synergistic effect that suggests that tACS may reinforce the effects of existing therapies by enhancing striatal dopamine receptor sensitivity.

From a pathophysiological point of view, the dual modulatory effect of tACS-i.e., simultaneous enhancement of corticospinal excitability and improvement of intracortical inhibition-distinguishes it from conventional symptomatic treatments. Of particular interest is the fact that this improvement is significantly associated with the modulation of neural oscillations in the beta band (13–30 Hz), which provides a new perspective for understanding the role of tACS in motor network reorganization. However, there is still no consensus among existing studies on the optimal stimulation frequency (*α*/*β*/*γ* bands), reflecting the heterogeneity of current evidence.

Based on these findings, future studies should prioritize multicenter phase III clinical trials with standardized 20 Hz β-band stimulation parameters (1.5 mA, 30 min/session) and incorporate long-term follow-up of at least 6 months. At the same time, optimizing the stimulation regimen by incorporating individualized EEG characteristics will help to achieve a paradigm shift from “one size fits all” to precise neuromodulation. Only through these systematic efforts can tACS truly become a scalable adjunct in the comprehensive treatment of Parkinson’s disease.

## Data Availability

The original contributions presented in the study are included in the article/Supplementary material, further inquiries can be directed to the corresponding authors.
