# Supplementary material for: Effects of transcranial alternating current stimulation on neurophysiologic motor function in Parkinson’s patients: a systematic review and meta-analysis
Source: Front Aging Neurosci. 2025 Sep 3;17:1621052. doi: 10.3389/fnagi.2025.1621052 (PMC12442322; doi:10.3389/fnagi.2025.1621052)
Supplement: Supplementary file 2 [file Table_2.DOCX]

**Table S2. The detailed search strategy**

| **Electronic databases** | **Search** | **Search strategy** |  | **Results** |
| --- | --- | --- | --- | --- |
| **PUBMED** | #1 | ("transcranial alternating current stimulation"[Title/Abstract] OR "tACS"[Title/Abstract]) AND ("Parkinson Disease"[MeSH Terms] OR ("idiopathic Parkinson's disease"[Title/Abstract] OR "lewy body Parkinson's disease"[Title/Abstract] OR " Parkinson's disease idiopathic"[Title/Abstract] OR " Parkinson's disease lewy body"[Title/Abstract] OR "paralysis agitans"[Title/Abstract] OR " Parkinson's disease"[Title/Abstract] OR "idiopathic parkinson disease"[Title/Abstract] OR "lewy body parkinson disease"[Title/Abstract] OR "primary parkinsonism"[Title/Abstract] OR "parkinsonism primary"[Title/Abstract] OR "parkinson disease idiopathic"[Title/Abstract])) |  | 43 |
| **EMBASE** | #1 | Title, abstract or author-specified keywords (Parkinson Disease OR Lewy Body Parkinson's Disease OR Parkinson's Disease, Idiopathic OR Parkinson's Disease, Lewy Body OR Paralysis Agitans) AND (transcranial alternating current stimulation OR tACS) |  | 40 |
| **Cochrane Library** | #1 | (Parkinson Disease OR Idiopathic Parkinson's Disease OR Lewy Body Parkinson's Disease OR Parkinson's Disease, Idiopathic OR Parkinson's Disease, Lewy Body OR Paralysis Agitans OR Parkinson's Disease OR Idiopathic Parkinson Disease OR Lewy Body Parkinson Disease OR Primary Parkinsonism OR Parkinsonism, Primary OR Parkinson Disease, Idiopathic):ti,ab,kw |  |  |
|  | #2 | (transcranial alternating current stimulation OR tACS) :ti,ab,kw |  |  |
|  | #3 | #1 and #2 |  | 58 |
| **Web of Science** | #1 | TS=(Parkinson Disease OR Idiopathic Parkinson's Disease OR Lewy Body Parkinson's Disease OR Parkinson's Disease, Idiopathic OR Parkinson's Disease, Lewy Body OR Paralysis Agitans OR Parkinson's Disease OR Idiopathic Parkinson Disease OR Lewy Body Parkinson Disease OR Primary Parkinsonism OR Parkinsonism, Primary OR Parkinson Disease, Idiopathic) AND TS=(transcranial alternating current stimulation OR tACS) |  | 4 |
